# Modified and Grafted Coronectomy: A New Technique and a Case Report with Two-Year Followup

**DOI:** 10.1155/2013/914173

**Published:** 2013-04-22

**Authors:** Michael Leizerovitz, Olga Leizerovitz

**Affiliations:** UCLA School of Dentistry, 10833 Le Conte Avenue, Los Angeles, CA 90095-1668, USA

## Abstract

*Purpose*. A standard coronectomy (intentional partial odontectomy) is recommended for mandibular third molar (MTM) extraction cases with a high risk of inferior alveolar nerve injury (IANI). However, complications such as inadvertent intraoperative root removal, post-op root migration, second molar (MSM) periodontal defects and others do exist. This report presents a new technique, the Modified and Grafted Coronectomy (MGC), describes the measures to prevent or minimize the known drawbacks of the standard coronectomy, and reviews the literature for comparison with three other IANI-prevention techniques. *Materials and Methods*. MGC was performed on two MTMs with nerve involvement and severe periodontal pockets on the distal of MSM. Modifications were: stabilizing the root stump to prevent intraoperative movement, creation of a large intrabony space for bone graft material, and grafting for periodontal healing while minimizing the possibility of post-op root migration. *Results*. Excellent overall periodontal improvement, with probing depths reduced to 3-4 mm. Panoramic radiograph displayed remarkable bone regeneration. No residual root migration was evident at the two year follow up. *Conclusion*. MGC may be a good alternative, especially in cases with periodontal defects on the distal of MSM. It may also help to minimize inadvertent intraoperative root removal and postoperative root migration.

## 1. Introduction

The removal of impacted mandibular third molar (MTM) in close proximity to the mandibular canal has proven to be problematic. One of the more severe risks is inferior alveolar nerve injury (IANI). The prevalence of this type of nerve injury in the twenty-four prospective studies reviewed varies between a minimum of 0% [1, 2] and a maximum of 8.4% [3]. The need to prevent these kinds of injuries is especially important since current treatment modalities of neurosensory deficit management (four surgical and two nonsurgical [4]) show only limited improvement in sensation. According to studies, complete recovery is uncommon with all types of available treatments [4, 5].

Coronectomy is the oldest and the best researched of the IANI-risk reducing procedures [6–19]. First described in 1984 [14], it has been extensively reviewed in multiple articles and retrospective studies, has been examined in eight prospective trials (Table 1), and has been now listed as a standard treatment option for surgical management of third molars by AAOMS ParCare 2012 in USA [20]. Despite those facts, coronectomy has not yet been accepted by the majority of the oral surgery community.

In the regular coronectomy, the aspect of the third molar root/s in the closest proximity to the inferior alveolar nerve (IAN) is intentionally retained. The clear benefit of a successful coronectomy is the avoidance of IANI. The disadvantages of this technique include deep periodontal pockets on the distal of the second molars (similar to those after extractions in comparable circumstances), root migration with the possible need of a second procedure, dry sockets, local postoperative infections, postoperative pain and inadvertent root removal, or root walk-out during surgery which may increase the risk of IANI (also known as a failed coronectomy) [6, 7, 17, 21, 22].

Deep pockets and other periodontal damages have been previously reported on the distal of the second molar teeth after third molar removal [23–26]. The measurements used in these studies were attachment levels, pocket depth and/or alveolar bone height. Shallower pocket depths postoperatively have been obtained through the debridement of the distal root of the second molar [27, 28]. Grafting following extraction has also minimized the development of second molar periodontal defects in the high-risk group (age ≥ 26 years., preexisting attachment loss ≥ 3 mm, and mesioangular or horizontal impaction) [29, 30] and in the younger patients group (ages 21–26 years) [31]. However, coronectomy in conjunction with grafting and distal root surface of the second molar debridement and demineralization has not been previously reported.

This paper provides a description of a case of a Modified and Grafted Coronectomy (MGC), developed by the authors. The goals of the technique are to decrease the incidence of intraoperative root walkout, to minimize the potential and/or preexisting periodontal pockets distal to the second molar similar to the “Preservation of periodontal health of adjacent teeth” Specific Therapeutic Goal of ParCare 2012 [20], and to decrease the risk of delayed root migration with the possible need for a second surgical procedure, all while preserving the excellent IANI-prevention record of a standard coronectomy. The reduction in pocket depth may also prevent or minimize the need for future periodontal interventions. The following case has met the goals described herein.

## 2. Clinical Report

A 37-year-old female, a smoker for the past 15 years but otherwise in good general health, was repeatedly referred for extractions of her MTM due to severe localized periodontal disease at the distal of the mandibular second molar (MSM) teeth. She stated that over the last four years she had consulted five oral surgeons who each suggested third molar extraction. The patient was also advised that she would have a higher than normal chance of bilateral permanent paresthesia. She reported that her periodontist warned her about the deteriorating periodontal health of both of her lower second molar teeth. She also reported occasional pressure and intermittent pain associated with the lower right third molar. 

**Figure 1:**
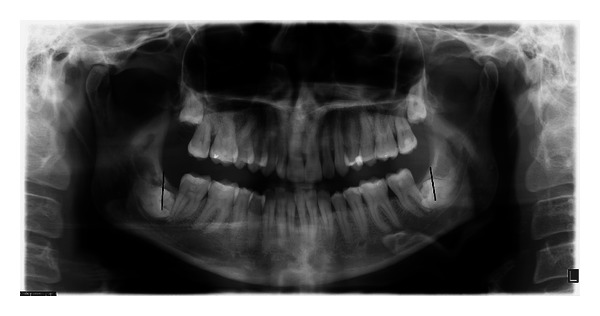
Preoperative panoramic radiograph. The direction of the initial cuts is marked.

At her consultation appointment, the patient presented with a panoramic radiograph and multiple dental treatment plans, all of which included extraction of the MTMs. A new panoramic radiograph was taken (Figure 1) which confirmed bilateral complete bony, horizontally impacted MTMs, Pell and Gregory's [32] class 2C on the lower right and 3C on the lower left with the roots in intimate radiographic proximity to the IAN bundle. The periodontal ligament space appeared sclerotic. The distal of the right MSM was tender to palpation and slightly swollen, which the patient stated had been bothersome for the last few weeks. Immediately prior to the surgical procedure, the periodontal probing depths were measured at 13 mm on the distal of right MSM and 8 mm on the distal of left MSM.

The patient was given the options and risks of observation, extractions with grafting, and Modified Grafted Coronectomy. The patient chose to have bilateral MGC performed. The treatment plan was reviewed and her consent was obtained.

**Figure 2:**
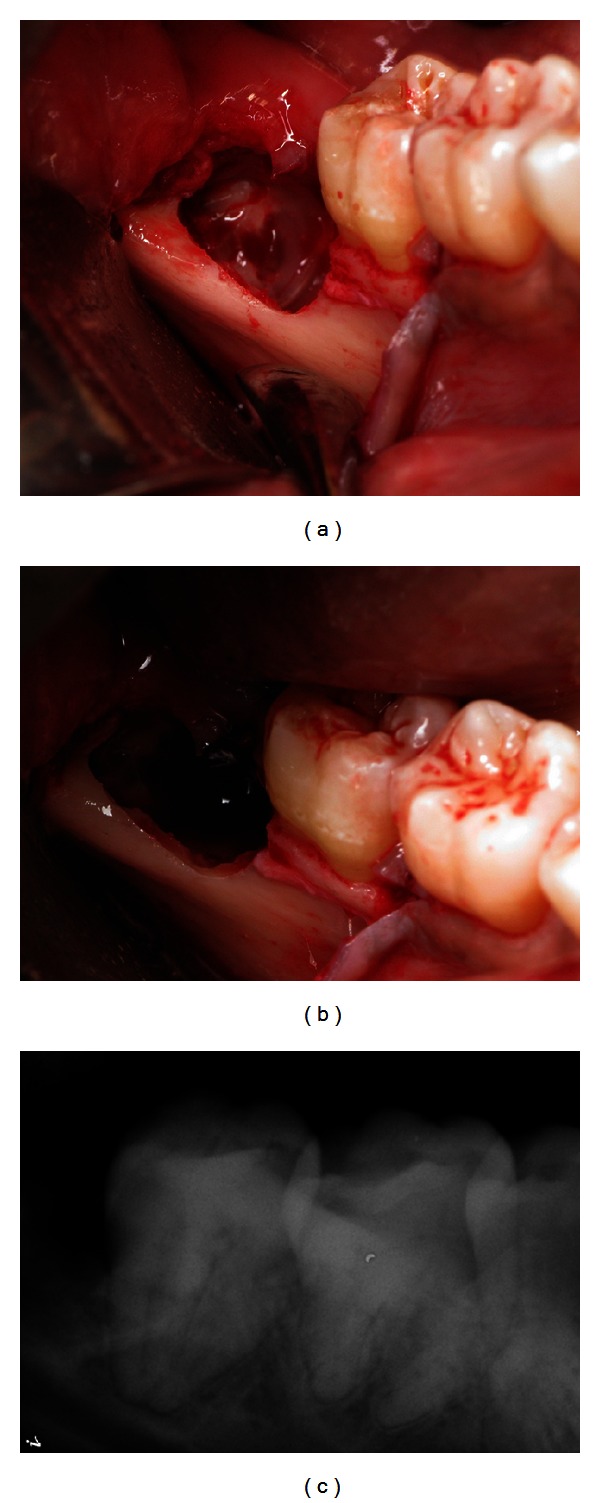
Lower right MTM: (a) after amputation of the crown, rotary instruments were used to reduce the distoocclusal section of the remaining stump; (b) after an additional cut and sectioning of the mesial portion of the tooth; (c) sufficient clearance to the second molar confirmed.

**Figure 3:**
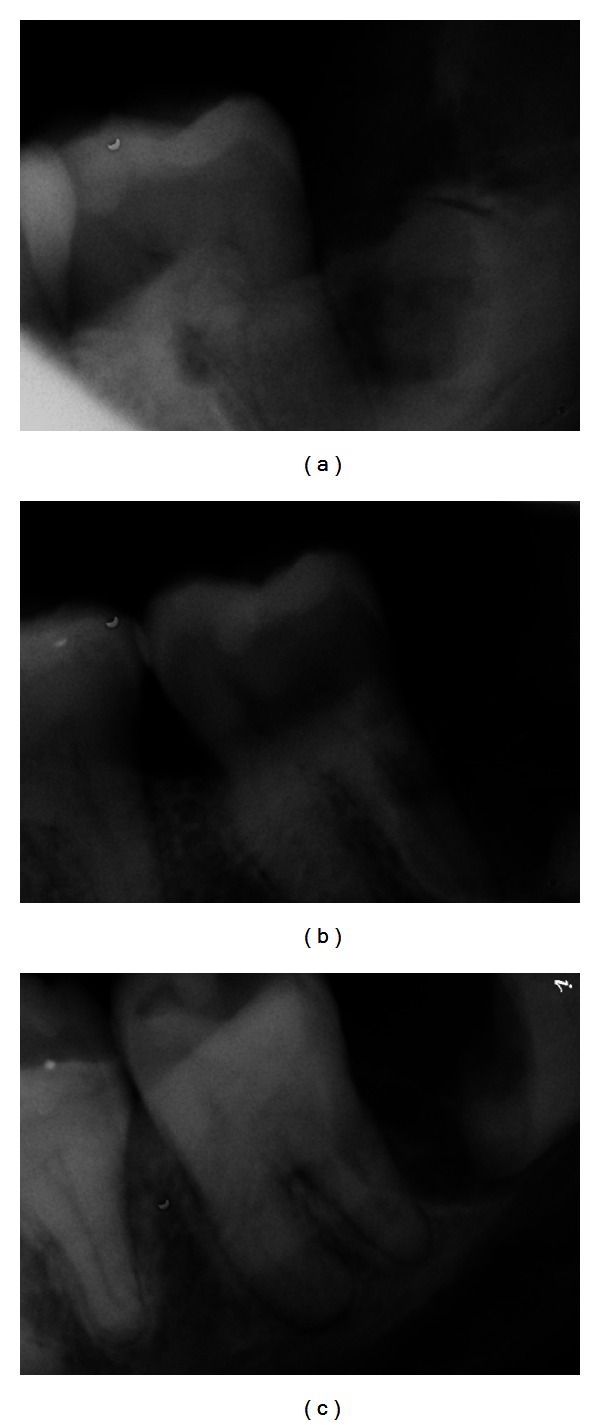
Lower left MTM: (a) after amputation of the crown; (b) after reducing the distoocclusal section of the remaining stump; (c) after an additional cut and removal of the mesial portion of the tooth, to create sufficient clearance to the second molar.

Due to the presence of infection, she was placed on oral Clindamycin 150 mg, 40 tablets, QID, and 0.12% chlorhexidine mouth rinse twice a day beginning two days prior to the procedure. The surgery was performed with i.v. sedation and local anesthesia. A lower right full thickness distally released envelope flap was reflected. No lingual retraction was used. A bony window was created for access. An initial vertical cut with a #703 cross cut fissure carbide FG bur, 2.1 mm diameter was made above the CEJ and oriented at a 20° angle to the distal root of the second molar, to facilitate coronal fragment removal. The cut was completed at 3/4 of the tooth diameter to avoid cutting into the lingual wall and/or IAN and to avert possible lingual nerve injury or IANI. The crown was broken off, without excessive apical pressure. After the removal of the first fragment, rest seats were created in the root portion at each of the subsequent steps. These were used to apply gentle apical pressure on the distal section with the sharp end of a Molt 9A periosteal elevator while grinding, cutting, and fracturing off the coronal fragments. This action stabilized the root portion, dampened the vibration, and helped to prevent inadvertent movement and therefore pressure of the remaining root against the IAN. Next, a #10 round carbide HP bur, 2.7 mm diameter was used to grind away the distoocclusal section of the remaining stump above the IAN, well below the bone crest level (Figure 2(a)). Due to insufficient clearance between the third molar stump and the second molar root, an additional cut was performed on the mesial portion of the remaining tooth, at 3/4 of the diameter and it was fractured off with a Schmeckebier #81 straight elevator (Figure 2(b)). A sufficient clearance to the second molar was confirmed with a radiograph (Figure 2(c)). The distal root of the second molar was detoxified and prepared for the bone graft by ultrasonic scaling and by a treatment with a 25% solution of citric acid for five minutes. A resorbable hydroxyapatite (HA) graft was placed into the bleeding site and no membrane was used. The flap was advanced for full coverage of the graft and primary closure and then sutured with 4-0 Vicryl sutures. The surgery on left MTM was carried out in a similar fashion. This side was somewhat more difficult due to less available space mesiodistally and significant resorption of the distal root of left second molar. (Figures 3(b) and 3(c)). Serial progress periapical radiographs were taken.

**Figure 4:**
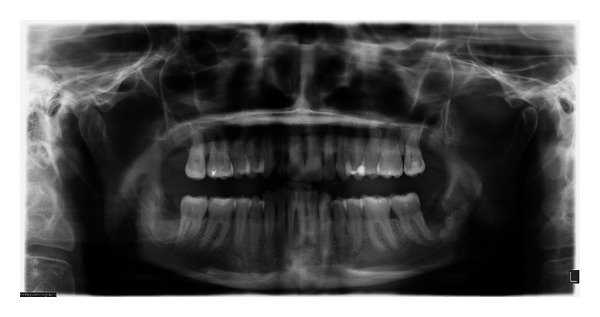
Six days postoperatively. Please note that the bone graft is radiolucent at this stage.

The patient was seen for a follow-up visit at six days postoperatively. The surgical sites appeared to be healing within normal limits. The absence of paresthesia in the IAN and lingual nerve distribution was assessed by the patient's subjective report of normal sensation with sharp, blunt, and light touch. A follow-up panoramic radiograph was obtained (Figure 4). 

Instructions were given for the patient to return for reassessment and radiographs every six months for the first two years, and once more after the third year. The patient was advised, if there are no problems found during the first three years, to return only if she experiences any unusual symptoms.

The patient and the referring dentist were advised to exercise special care with the grafted sites for the first six to nine months following treatment by not traumatizing them needlessly and to allow the graft to mature. This included no periodontal pocket charting, scaling on the distal of the second molars, and/or manipulating with any hard objects by the patient. According to the manufacturer, the grafted area becomes radiopaque in comparison to the surrounding bone indicating that the resorbable HA graft has matured.

## 3. Results

**Figure 5:**
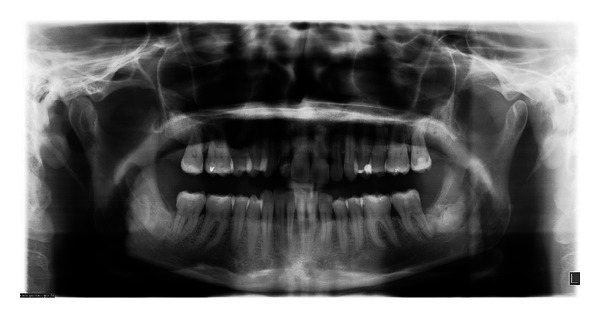
A 23-month follow-up with excellent healing.

At the two-year follow-up the patient stated the areas felt fine with no sensitivity around the second molars. Periodontal examination revealed decreased periodontal pocket depths, down to three and four mm. Panoramic radiograph was obtained (Figure 5), displaying good bone fill with excellent periodontal improvement overall. No residual root migration was evident. 

## 4. Discussion

Regular coronectomy is the best studied alternative to the extraction of teeth, which are at a high risk for IANI, as determined by radiographic signs [6, 7, 10, 17, 33–37]. It has now been accepted in USA as a standard and is no longer a controversial treatment option at Third Molar Multidisciplinary Conference in Washington, DC, on October 19, 2010, and AAOMS ParCare 2012 [20, 38]. There are several technique variations to perform a standard coronectomy procedure [6, 7, 9, 10, 14, 16, 17]. While nerve damage is avoided in a successful standard coronectomy, other complications can arise. 

IANI was the most serious complication which occurred during some failed coronectomies, where the remaining root was inadvertently mobilized during surgery. This mandated the surgeons to proceed with the extraction of the entire root in 4–38% of the cases [7–9, 17], also resulting in temporary IANI, with an incidence of 8.3–11.1% [7, 8].

Other known complications are deep periodontal pockets on the distal of the second molar, delayed postoperative root migration with the possible need of a second procedure, postoperative pain, dry socket, and infection [7–9, 21, 22].

These drawbacks could be a possible motivation for the recent emergence of additional IANI-risk reducing procedures: staged removal (the removal of a portion of the crown, creating space for the root to erupt away from the IAN, with subsequent removal of the remainder of the root) [39], pericoronal ostectomy (the removal of the overlying bone to allow for the tooth to erupt away from the IAN) [21], and orthodontic extractions (active orthodontic movement of the tooth away from the IAN) [40, 41]. Pericoronal ostectomy and orthodontic extraction allow for bone formation on the distal of the second molar which occurs due to the occlusal extrusion as the MTM moves away from the IAN [21, 41]. While it is obvious that preservation of periodontal health, regenerating bone and preventing or reducing the further need of periodontal surgery at the distal of the lower second molar, is an important advantage for the patients [20], a standard coronectomy procedure lacks this benefit. 

With only one recorded case of permanent IANI in its history [17], there is an abundance of evidence that coronectomy has had great success in avoiding IANI. While performing the standard coronectomy since 1995, we have experienced both its IANI avoidance benefits as well as all its negative side effects. In an effort to overcome these, we attempted various modifications to the different steps of the original technique. 

Ultimately, the technique used in the case reported above appears to have the most encouraging outcome. In it we introduced a series of amendments to the standard coronectomy procedure and called it a Modified and Grafted Coronectomy. 

As with the standard coronectomy, MGC involves the removal of the crown and part of the root/s of an impacted MTM in cases with a high risk of IANI. This modified procedure introduces steps to prevent the complication of inadvertent intraoperative root loosening. It accomplishes this by stabilizing the radicular fragment during cutting as well as when separating the coronal section off, thus overall decreasing the risk of nerve injury. Yet as another modification, to reduce or prevent periodontal pockets on the distal of the second molar, the technique calls for the creation of periodontal “scaffolding,” which is achieved through grafting, thus the name Modified and Grafted Coronectomy. 

Scaling and debridement [27, 28] as well as root surface demineralization [42–44] with either citric acid or tetracycline of the distal of the second molar were the necessary elements for the reattachment success in all our cases. Of the graft materials we used, only resorbable HA (with or without use of resorbable collagen membrane) or cortical allograft with the membrane gave us satisfactory results.

In our experience with grafting, it is important to create a larger vertical space to allow for bone regeneration. According to Hatano “even if appropriate trimming 2 to 3 mm below the alveolar crest is done, bone coverage on the resected surface is sometimes delayed because of significant root migration” [10]. Combining grafting with the creation of a larger space resulted in a bulkier graft, which worked well in this case. The resulting periodontal healing is very encouraging (Figure 5), as well as the absence of root migration which in turn averts the possible necessity of a second surgical procedure to remove the root fragment. The findings in this paper appear to be promising without the side effects of the standard coronectomy procedure. 

MGC appears to be an alternate technique that could be considered for nearly all coronectomy cases of vertical, mesioangular, or horizontal impactions and especially in the cases which have preexisting periodontal lesions or are expected to have large postsurgical defects. A possible additional benefit is the potential prevention for the need of further pocket reduction surgery on the distal of the lower second molar, minimizing the need of subsequent treatments and visits.

Possible considerations of the technique are the additional costs of the graft and the time required to perform the procedure. Further modifications and enhancements could include the use of different graft materials and/or the use of a membrane. Additional research with a large cohort of patients and randomized prospective studies are needed to verify the outcome. 

While more research is absolutely necessary, this new technique and/or just some of its steps appears to offer a viable alternative for a coronectomy practitioner, helpful in obtaining superior results in addition to the existing IANI-risk reduction procedures.

## Figures and Tables

**Table 1:** Published prospective trials.

Reference	Author	Article	Published in journal	Year published	*n* subjects	*n* teeth	Study design
[6]	Pogrel et al.	“Coronectomy: a technique to protect the inferior alveolar nerve”	J Oral Maxillofac Surg	2004	41	50	Prospective cohort
[7]	Renton et al.	“A randomised controlled clinical trial to compare the incidence of injury to the inferior alveolar nerve as a result of coronectomy and removal of mandibular third molars”	Br J Oral Maxillofac Surg	2005	128	94	Randomized controlled trial
[8]	Pogrel	“An update on coronectomy”	J Oral Maxillofac Surg	2009	Not available	450	Prospective cohort
[9]	Leung and Cheung	“Safety of coronectomy versus excision of wisdom teeth: a randomized controlled trial”	Oral Surg Oral Med Oral Pathol Oral Radiol Endod	2009	231	171	Randomized controlled trial
[10]	Hatano et al.	“Clinical evaluations of coronectomy (intentional partial odontectomy) for mandibular third molars using dental computed tomography: a case-control study”	J Oral Maxillofac Surg	2009	220	102	Case control study
[11]	Dolanmaz et al.	“A preferable technique for protecting the inferior alveolar nerve: coronectomy”	J Oral Maxillofac Surg	2009	43	47	Prospective cohort
[12]	Cilasun et al.	“Coronectomy in patients with high risk of inferior alveolar nerve injury diagnosed by computed tomography”	J Oral Maxillofac Surg	2011	120	175	Prospective cohort
[13]	Leung and Cheung	“Coronectomy of the lower third molar is safe within the first 3 years”	J Oral Maxillofac Surg	2012	98	135	Prospective cohort
